# Mastering the Massive: The Surgical Strategy and Outcomes in a Case of a Large Breast Hamartoma

**DOI:** 10.7759/cureus.73896

**Published:** 2024-11-18

**Authors:** Priya P Ahire, Ami S Gandhi, Yogesh Jaiswal, Ashwini Binorkar, Pritesh N Joshi, Gayathri Sukumaran

**Affiliations:** 1 General Surgery, Grant Government Medical College and Sir JJ Group of Hospitals, Mumbai, IND; 2 Plastic and Reconstructive Surgery, Grant Government Medical College and Sir JJ Group of Hospitals, Mumbai, IND; 3 Surgery, Grant Government Medical College and Sir JJ Group of Hospitals, Mumbai, IND; 4 Pathology, Grant Government Medical College and Sir JJ Group of Hospitals, Mumbai, IND

**Keywords:** benign breast condition, breast disease, breast hamartoma, desmin positive, diagnostic mammography, fibroadenolipoma, lattismus dorsi flap, mri breast, pseudocapsule, reconstructive breast surgery

## Abstract

Hamartomas are rare, benign pseudotumors consisting of a mixture of ducts, lobules, fibrous stroma, and adipose tissue. Despite their benign nature, these lesions can present significant clinical challenges and may be underrecognized. A 48-year-old female presented with a progressively enlarging lump in the right breast over eight years. The lump, measuring 42 x 45 cm, displaced the nipple-areola complex and exhibited a bosselated, variegated consistency. Magnetic resonance imaging revealed a well-defined, lobulated lesion (15.3 x 21 x 26 cm) involving the right breast parenchyma and skin surface. Histopathology from core biopsies showed predominantly fibrocollagenous tissue with mild atypia. The patient underwent wide local excision and reconstruction with a latissimus dorsi myocutaneous flap. Postoperative histopathology confirmed a well-encapsulated mammary hamartoma (29 x 25 x 11.5 cm, 6 kg) with no malignant features. Hamartomas are usually benign and can be challenging to diagnose due to their complex, disorganized architecture. Imaging modalities such as mammography, ultrasound, and MRI are essential for diagnosis, while histopathological examination confirms the nature of the lesion. Surgical excision remains the definitive treatment, addressing both functional and cosmetic concerns. Although malignancy is rare, precise diagnosis and thorough surgical management are critical. This case is the largest breast hamartoma documented in the literature to our best knowledge, providing significant insights into its clinical presentation and management.

## Introduction

In 1981, hamartomas were officially recognized in the WHO classification. They are rare entities described as well-circumscribed pseudotumors containing lobules and ducts surrounded by varying amounts of connective tissue, which can be fibrotic or adipose [1]. They are also referred to as fibroadenolipoma, lipofibroadenoma, or adenolipoma [2]. Breast hamartoma is a rare and frequently underrecognized benign lesion, constituting about 4.8% of all benign breast masses [3]. Hamartomas are slow-growing lesions typically measuring 2-5 cm in diameter, though they can occasionally grow to larger sizes as seen in this case presentation [4]. Herbey presented a case of a 48-year-old female with a giant breast hamartoma, which was managed by surgical excision followed by reconstruction.

## Case presentation

A 48-year-old female presented with a primary complaint of a lump in the right breast, which had progressively increased over the past eight years. There was no history of a similar lump elsewhere in the body, nor any complaints of nipple discharge, pain, fever, or associated redness. She had no history of significant weight loss. The patient had a history of thermal burns involving the chest, neck, bilateral upper limbs, and lower part of the face 27 years prior, for which she underwent skin grafting. Her medical and family history was unremarkable.

**Figure 1:**
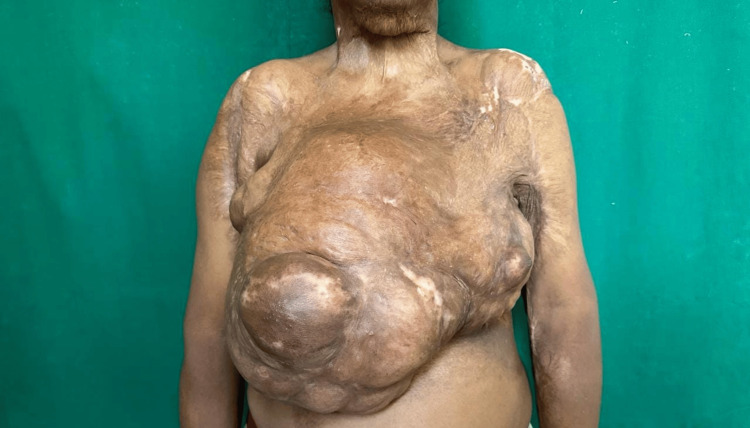
Clinical picture of right breast lump with distortion of right nipple areola complex and skin contractures over chest, neck, and arm

**Figure 2:**
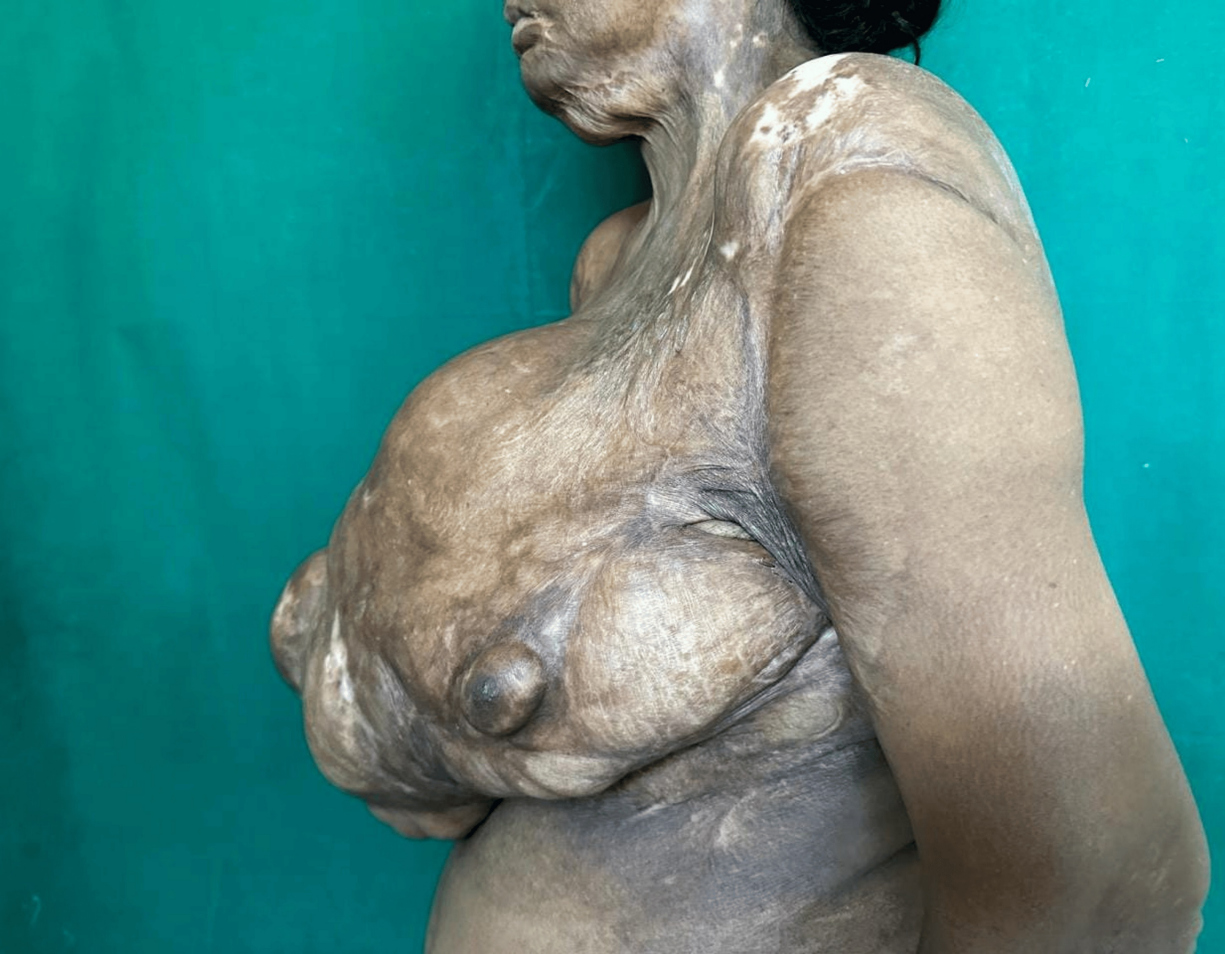
Clinical picture of lateral view of right breast lump with bosselated appearance and skin contractures and displaced left nipple areola complex

On examination, a solitary breast lump of size 42 cm x 45 cm is present over the right breast, encompassing all the quadrants, distorting the nipple-areola complex (NAC) of the right side, and displacing the NAC of the opposite breast laterally and superiorly as seen in Figures 1-2. There were hypertrophic scars and contractures present over the neck, chest region, bilateral axilla, and elbow. The lump had a bosselated appearance and was variegated in consistency. The lump was not fixed to the underlying structure. The patient had no evidence of axillary lymphadenopathy.

**Figure 3:**
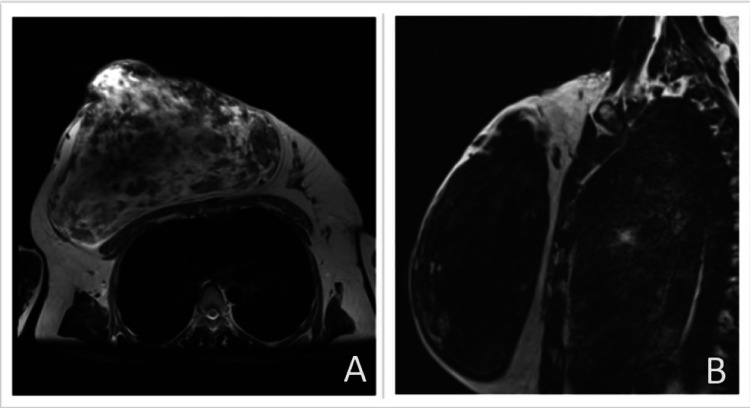
MRI image showing a well-defined, lobulated, heterogeneous lesion of size 15.3 x 21 x 26 cm containing both fat and soft-tissue density. Image A shows an axial view of a thin, radiopaque pseudocapsule suggestive of a fibroadenolipoma (hamartoma) in the left breast, depicting a breast-within-a-breast appearance. Image B shows a sagittal view of the right breast

**Figure 4:**
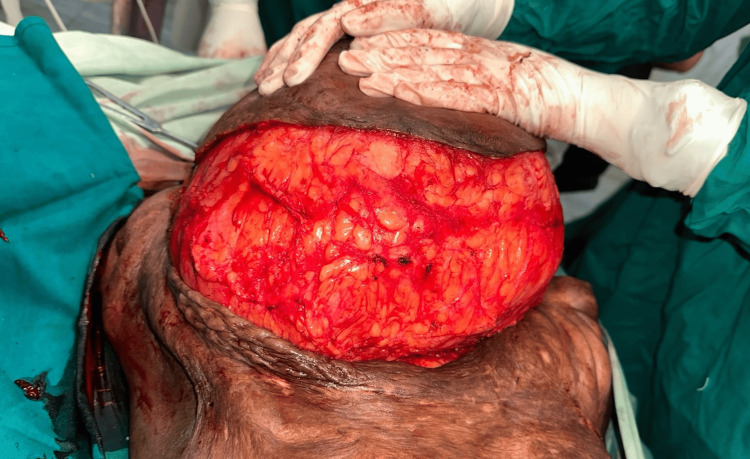
Intraoperative image of right breast giant hamartoma after dissecting from surrounding tissue

**Figure 5:**
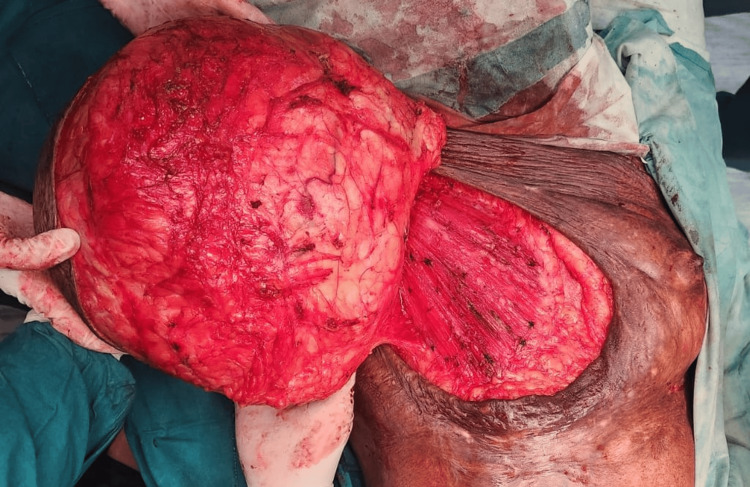
Intraoperative image of giant right breast lump after dissecting it from pectoralis major muscle

**Figure 6:**
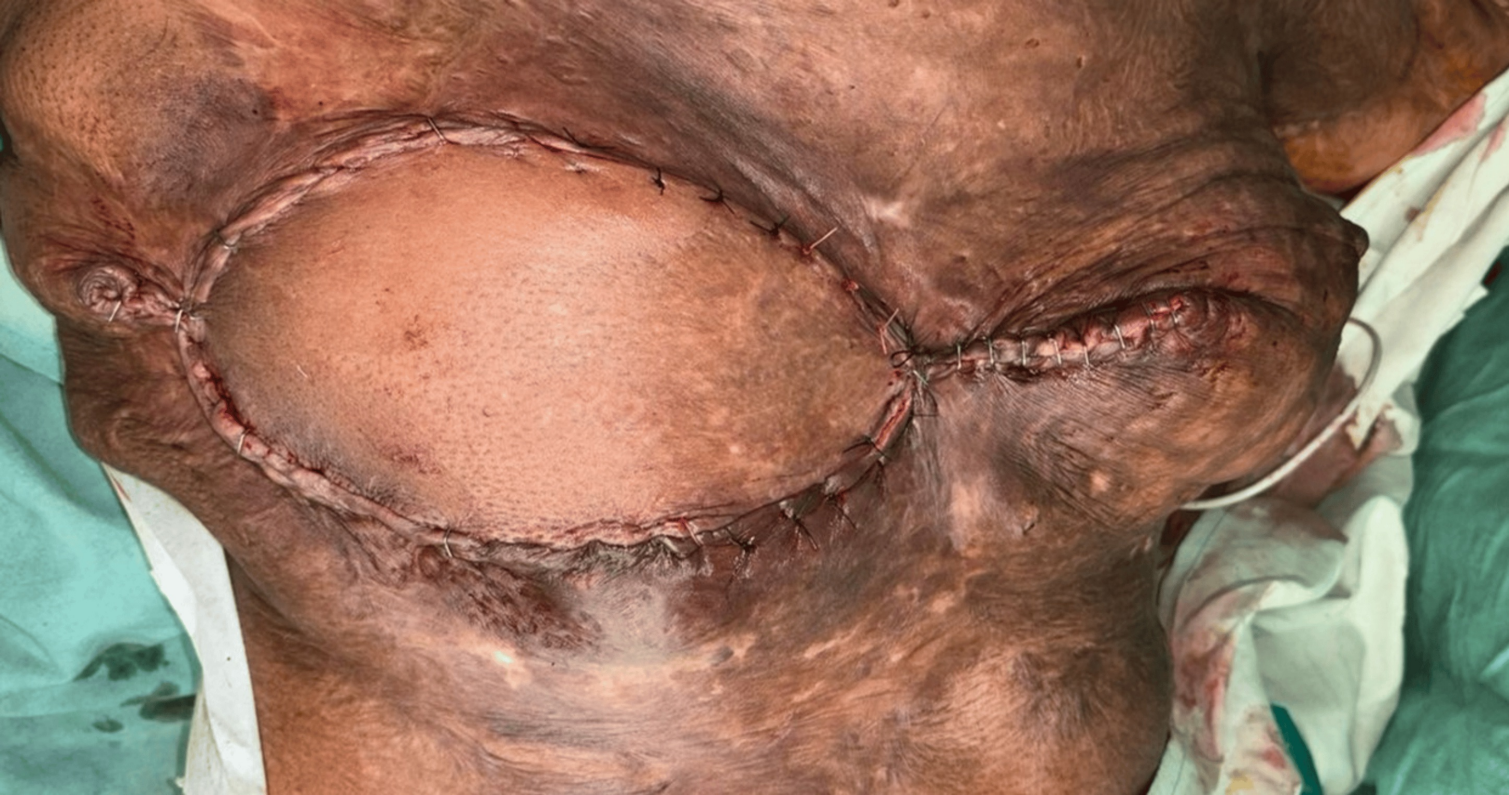
Postoperative image showing the stapler line following a wide local excision of a right breast hamartoma, accompanied by breast reconstruction using a latissimus dorsi myocutaneous flap

Further investigation with magnetic resonance study revealed a well-defined, lobulated, heterogeneous lesion of size 15.3 x 21 x 26 cm completely replacing the right breast parenchyma, extending into the skin surface with involvement of the nipple-areola complex with no lymphadenopathy. The lesion exhibits a well-defined margin, without encroachment or infiltration into adjacent structures, and has a low suspicion of malignancy classified as BIRADS IVa as seen in Figure 3. Multiple trucut biopsies taken from all four quadrants of the breast revealed predominantly fibrocollagenous tissue along with mild atypia with anisonucleosis of focal ductal epithelial cells. The patient underwent wide local excision of the lump as the histopathology report did not suggest malignancy as seen in Figures 4-5. The remaining defect was reconstructed by using a latissimus dorsi myocutaneous flap from the back region in the same setting as seen in Figure 6. The excised specimen weighed 6 kg. The patient tolerated the procedure well with no intraoperative or postoperative complications.

**Figure 7:**
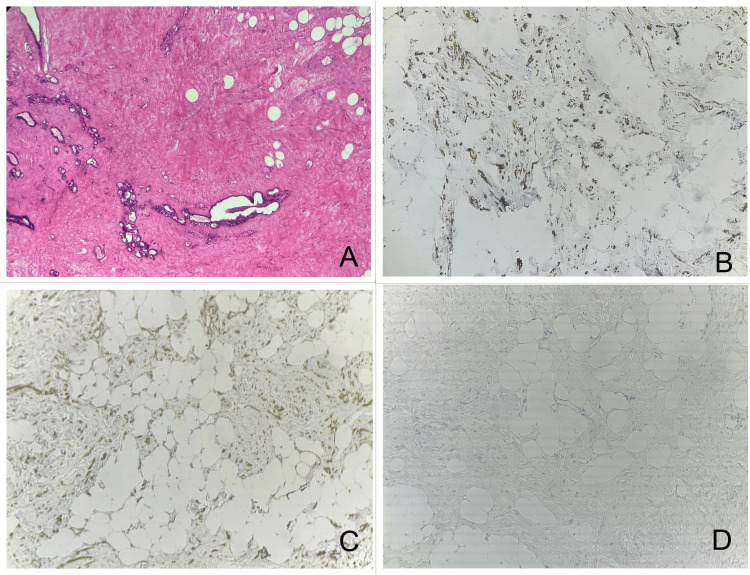
Microscopic examination of breast hamartoma. Image A: H&E slide depicting a hypocellular lesion characterized by widely spaced, uniform spindle cells embedded in a dense collagenous stroma with no evidence of atypia or mitotic activity. Image B: Immunohistochemistry slide showing desmin positivity. Image C: Immunohistochemistry slide showing SMA positivity. Image D: Immunohistochemistry slide showing pancytokeratin negativity

Histopathological examination revealed a well-encapsulated tumor measuring 29 x 25 x 11.5 cm with an unidentifiable nipple-areola complex of the right side, greyish and glistening. On microscopy, spindle cells, adipose tissue with collagenized stroma, and focal myxoid areas are seen in the background with entrapment of skeletal muscles in the periphery as shown in Figure 7. There was no evidence of atypia or mitotic activity. The immunohistochemistry report was positive for smooth muscle actin (SMA), desmin, and progesterone receptor (PR) and immunonegative for estrogen receptor (ER), pancytokeratin, S-100, and CD 34. Benign breast ductular elements are seen along with abnormal elastic fibers. The final diagnosis was consistent with mammary hamartoma. The patient was followed up for six months and had no complications.

## Discussion

Hamartomas are common in middle-aged women during the perimenopausal period [4]. These lesions can develop in various parts of the body, with common sites being the lungs, skin, hypothalamus, spleen, and kidneys, with the lungs being the most frequent location [5]. Hamartomas can be diagnosed definitively using mammography, ultrasonography (USG), magnetic resonance imaging (MRI), fine-needle aspiration biopsy (FNAB), and core biopsy [6]. Clinically they are painless, mobile, soft to firm lumps [7]. On ultrasound, hamartomas typically present as well-circumscribed masses with smooth borders and can exhibit hypoechoic or heterogeneous echogenicity. They generally do not show the retrotumor acoustic phenomenon, which is a distinguishing feature used to differentiate benign from malignant lesions. On mammography, the appearance of hamartomas can vary depending on the proportion of their component tissues. Typically, they are seen as non-homogeneous structures with dense nodules composed of fibrous tissue, surrounded by a thin radio-opaque pseudocapsule formed by the displacement of surrounding breast parenchyma [7]. Hamartomas are treated by surgical excision. This lesion can be easily underestimated if clinical findings of a distinct lump or breast asymmetry, along with imaging features, are not thoroughly evaluated.

On microscopic examination, hamartomas are circumscribed masses characterized by a mixture of ducts, lobules, fibrous stroma, and adipose tissue in varying proportions, often with a disorganized architectural pattern [8]. They may also include additional components such as smooth muscle, cartilage, or pseudoangiomatous stromal hyperplasia (PASH). A specific type of hamartoma, known as a myoid hamartoma, exhibits prominent myoid changes. Additionally, there are two variants of hamartomas: adenolipoma and chondrolipoma [8]. Pathologists must remain vigilant for the possibility of coincidental epithelial malignancy within the lesion, as there is a potential risk of recurrence [9]. Breast hamartomas are not premalignant. However, given their glandular breast tissue, breast hamartomas can rarely undergo malignant changes similar to normal breast tissue. Therefore, achieving a definitive histopathological diagnosis is crucial. The incidence of malignancy in normal breast tissue within the hamartoma is as low as 0.1% [6]. Breast cancer patients harboring a germline PTEN mutation face an elevated risk of developing breast cancer along with endometrial, thyroid, renal, and colorectal cancers. Hence, it is advisable to implement enhanced screening for these associated cancers and conduct those in first-degree relatives as well [10]. In breast hamartomas, smooth muscle cells also express CD34, in addition to smooth muscle actin and desmin. The presence of hormone receptors (ER and PR) and CD34 positivity is linked to the derivation of these smooth muscle cells from undifferentiated stromal cells through a metaplastic process [11]. Tumor resection was indicated in this case to address the significant deformity of the right breast, which not only impacted the patient's physical appearance but also raised persistent diagnostic uncertainties. This intervention aimed to provide clarity regarding the underlying pathology while restoring the anatomy.

This case, based on current literature and as per existing reports, represents the largest breast hamartoma documented in the literature to date, highlighting the rarity and unique clinical presentation of such tumors. The findings contribute valuable insights into the management and characteristics of a giant breast hamartoma [11,12].

## Conclusions

Surgical excision, along with thorough histopathological evaluation, is essential for favorable outcomes and resolving diagnostic uncertainties. Despite its low incidence, breast hamartoma should be included in the differential diagnosis for atypical presentations. While mammographic findings may raise suspicion, surgical intervention is necessary when preoperative tissue diagnosis is inconclusive. This approach supports accurate diagnosis and helps in effective treatment planning.

## References

[1] Charpin C, Mathoulin MP (1994). Reappraisal of breast hamartomas. A morphological study of 41 cases. Pathol Res Pract.

[2] Guray M, Sahin AA (2006). Benign breast diseases: classification, diagnosis, and management. Oncologist.

[3] Kelkar R (2018). A case report of a large myoid hamartoma of breast. J Med Sci Clin Res.

[4] Sanal HT, Ersoz N, Altinel O, Unal E, Can C (2006). Giant hamartoma of the breast. Breast J.

[5] Murugesan JR, Joglekar S, Valerio D, Bradley S, Clark D, Jibril JA (2006). Myoid hamartoma of the breast: case report and review of the literature. Clin Breast Cancer.

[6] Tazeoğlu D, Dağ A, Arslan B, Berkeşoğlu M (2021). Breast hamartoma: Clinical, radiological, and histopathological evaluation. Eur J Breast Health.

[7] Tele JS, Patil NJ, Bhosale S, Gupta SO (2018). An unusual case of huge hamartoma of breast in a 23 year old female: a case report. Int J Res Med Sci.

[8] Agarwal I, Blanco L (2024). Hamartoma. https://www.pathologyoutlines.com/topic/breasthamartoma.html.

[9] da Silva BB, Rodrigues JS, Borges US, Pires CG, Pereira da Silva RF (2006). Large mammary hamartoma of axillary supernumerary breast tissue. Breast.

[10] Rumpf AL, Mathiak M, Schäfer FK (2021). A giant mammary hamartoma in a young breast cancer patient. Breast Care (Basel).

[11] Palo S, Agrawal V (2024). Giant Myoid mammary hamartoma: a case report. J Cancer Res Ther.

[12] Mahmoud W, El Ansari W, Hassan S, Alatasi S, Almerekhi H, Junejo K (2021). Giant mammary hamartoma in a middle aged female. Case report and review of literature of the last 15 years. Int J Surg Case Rep.

